# P-1028. Catheter Related Bloodstream Infection with Coagulase-Negative Staphylococci in Hematological Malignancy

**DOI:** 10.1093/ofid/ofaf695.1224

**Published:** 2026-01-11

**Authors:** Megan Biggs, Brian Lahr, Nischal Ranganath, Aaron J Tande

**Affiliations:** The Mayo Clinic, Rochester, MN; Mayo Clinic, Rochester, Minnesota; Mayo Clinic, Rochester, Minnesota; Mayo Clinic, Rochester, Minnesota

## Abstract

**Background:**

Catheter related bloodstream infections (CRBSI) are associated with increased resource use, treatment costs, and mortality among patients with cancer. Coagulase-negative staphylococci (CoNS) are the most common cause of central venous catheter (CVC) infection in this population. While CVC removal remains the gold standard, CRBSI with CoNS is sometimes managed with CVC salvage, despite incomplete safety and efficacy data in patients with hematological malignancy.

**Table 1.**
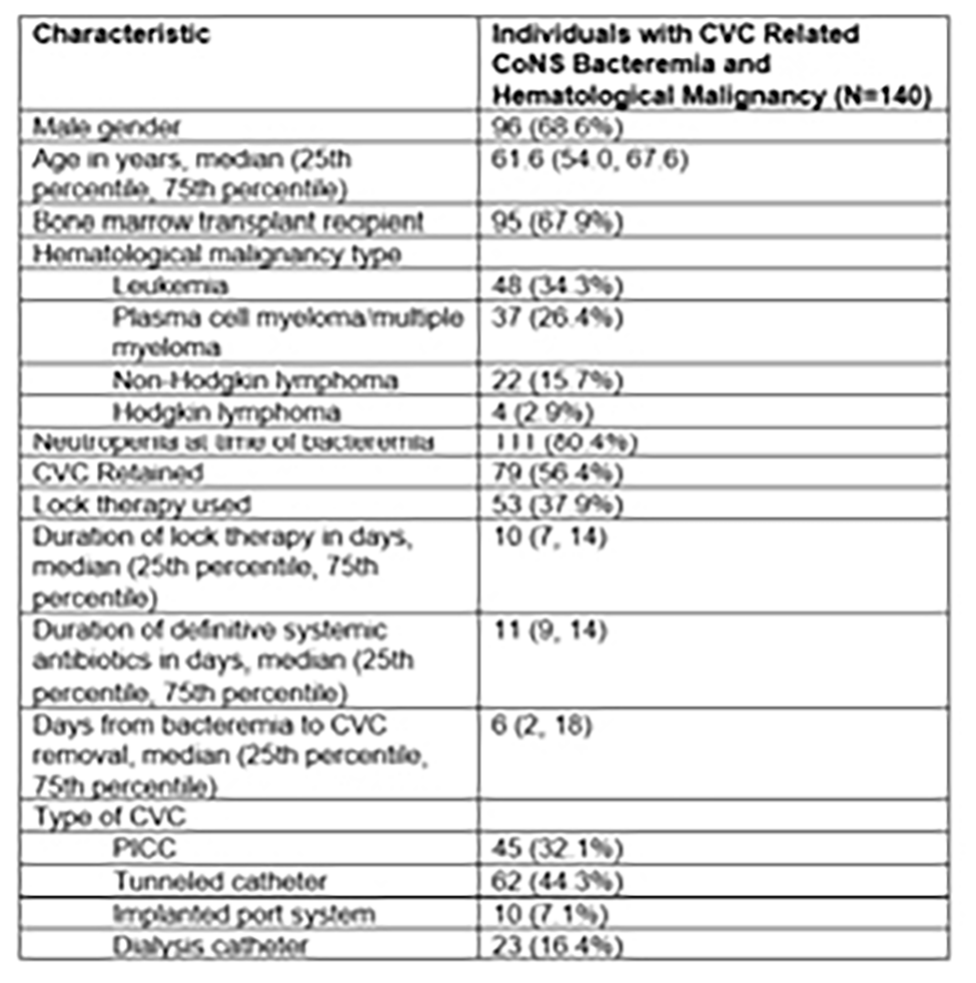
Baseline Demographic and Clinical Data

**Table 2.**
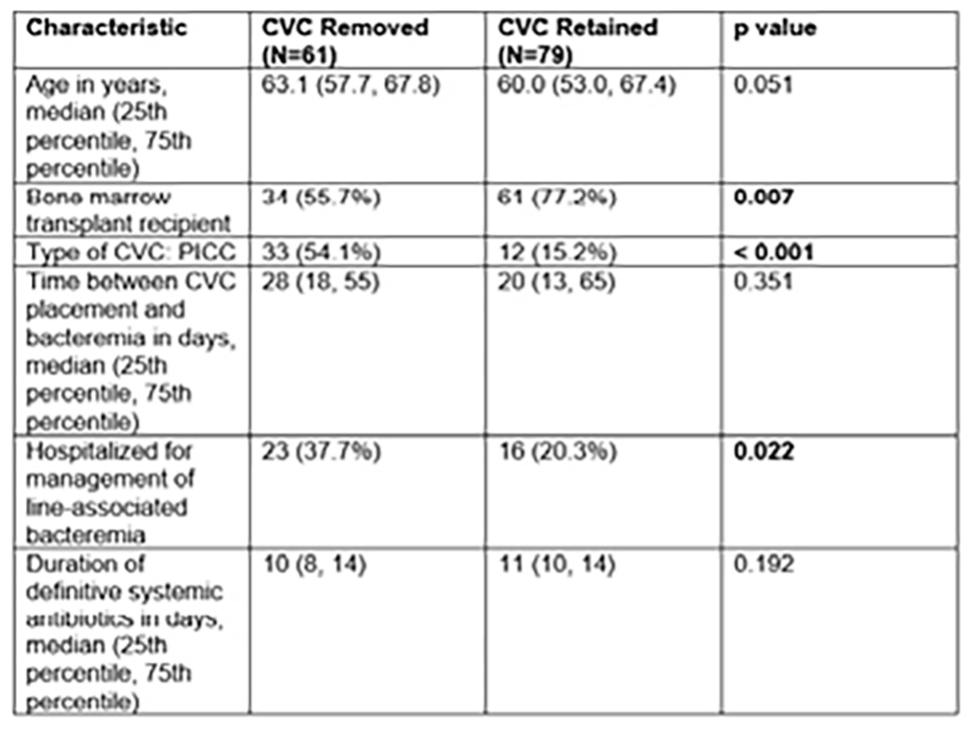
Baseline Characteristics by Treatment Strategy

**Methods:**

A retrospective review was conducted of adult patients at Mayo Clinic Rochester from 2013 to 2024 diagnosed with hematological malignancy and CRBSI with CoNS. Patients were included in a “CVC removed” group if the line was removed within 5 days of bacteremia onset, and all others were included in a “CVC retained” group. Electronic medical records were evaluated for relevant clinical comorbidities, risk factors, and management. The primary outcomes of interest were 90-day mortality and relapse. To control for baseline differences between groups, we employed a propensity weighted analysis based on inverse probability of treatment weighting using the propensity score.

**Figure:**
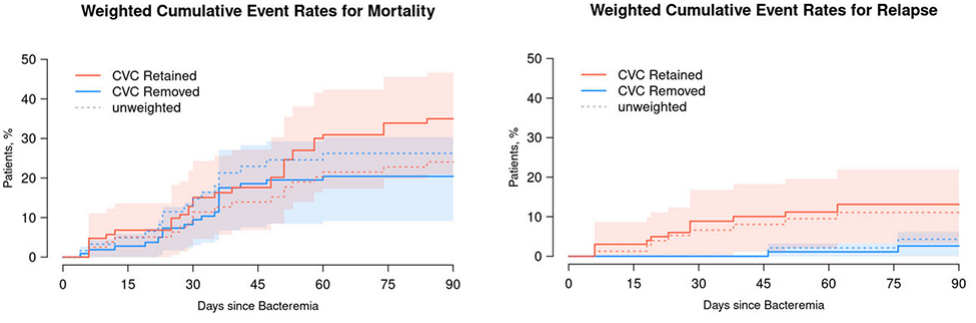
Weighted cumulative event rates for Relapse and Mortality

**Results:**

A total of 140 cases were included, of whom 95 (67.9%) had undergone BMT and 111 (80.4%) were neutropenic at time of CRBSI. The predominant CoNS identified was *Staphylococcus epidermidis* (88.6%). 79 (56.4%) patients had the CVC retained and 61 (43.6%) underwent CVC removal. Those in whom the CVC was retained were more likely to be BMT recipients (p=0.007). However, line removal was more common with PICC lines (p< 0.001) and in those hospitalized for management of line-associated bacteremia (p=0.022). Within 90 days, there were 10 patients (cumulative incidence 8.2%) who had microbiological relapse (2 [4.3%] in the CVC removed group and 8 [11.1%] in the CVC retained group). Propensity weighted analysis demonstrated significantly higher rate of microbiologic relapse with CVC retention (p=0.043) but no difference in mortality (p=0.128), as shown by weighted cumulative event rate curves in Figure 1.

**Conclusion:**

Despite the finding of increased risk of relapse associated with CVC retention, there was no significant difference in mortality between CVC removal and retention among individuals with CoNS CRBSI and hematological malignancy.

**Disclosures:**

All Authors: No reported disclosures

